# Self‐Assembly of Peptides and Biomolecular Systems Into Functional Nanomaterials

**DOI:** 10.1002/psc.70101

**Published:** 2026-05-06

**Authors:** Malak Fares, Othman Al Musaimi

**Affiliations:** ^1^ School of Pharmacy Newcastle University Newcastle upon Tyne UK; ^2^ Translational and Clinical Research Institute, Faculty of Medical Sciences Newcastle University Newcastle upon Tyne UK; ^3^ Department of Chemical Engineering Imperial College London London UK; ^4^ Orthogonal Peptides Limited London UK

## Abstract

Peptide self‐assembly represents a versatile and programmable strategy for generating functional nanomaterials with broad biomedical relevance. This review outlines the physicochemical principles governing assembly, highlighting cooperative noncovalent interactions, hydrogen bonding, π–π stacking, electrostatics and hydrophobic forces that drive hierarchical organisation into supramolecular structures. Key analytical techniques for characterising peptide assemblies and nanostructures are also summarised. The contribution of secondary structural motifs, particularly α‐helices and β‐sheets, is explored in relation to morphology, stability and biological function. α‐Helical coiled‐coil peptides form well‐defined nanotubular architectures suitable for cargo encapsulation, whereas β‐sheet peptides assemble into nanofibrillar networks and hydrogels with tuneable mechanical properties and sustained release profiles, as illustrated by systems such as RQDL10. Beyond peptides, protein and DNA self‐assembly further expand the biomolecular design space. Protein‐based systems leverage hydrophobic and Debye–Hückel electrostatic interactions to build hierarchical, functional architectures. DNA platforms enable programmable, stimulus‐responsive assembly, including enzyme‐ and logic‐controlled activation and hybridisation‐driven formation of reversible higher‐order nanostructures. Applications in drug delivery, tissue engineering and regenerative medicine are discussed alongside challenges such as limited in vivo stability, proteolytic degradation and scalability. Emerging approaches—including rational design, sequence engineering and advanced fabrication—aim to improve predictability and reproducibility, positioning biomolecular self‐assembly as a unified platform for next‐generation biomaterials.

## Graphical abstract

Peptide self‐assembly is driven by hydrogen bonding, electrostatic and hydrophobic interactions, forming α‐helical and β‐sheet nanostructures. These systems enable hydrogels, encapsulation and sustained release for biomedical use. Similar principles apply to protein and DNA assemblies, producing hierarchical, programmable, stimulus‐responsive nanomaterials for drug delivery, tissue engineering and regenerative medicine applications fields.

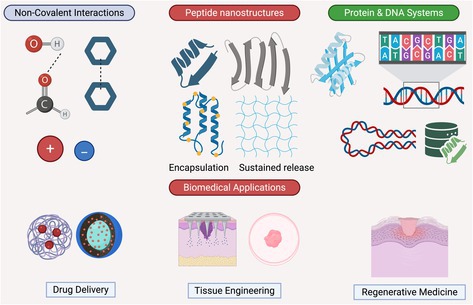

## 1 Introduction

Peptides, defined as short chains of up to 50 amino acids, perform diverse biological functions, including roles in cellular signalling and tissue repair [1, 2]. They are particularly attractive as building blocks for biomaterials due to their intrinsic biocompatibility, biodegradability and relative ease of synthesis and modification [3, 4]. Peptides are integral to a wide range of physiological processes, underpinning essential biological functions throughout the human lifespan. They contribute to both structural and regulatory roles, including muscle development, tissue repair and serving as fundamental components of hormones and neurotransmitters [5]. Beyond the canonical set of 20 natural amino acids, the incorporation of non‐natural amino acids further expands the structural and functional diversity of peptide systems, enabling precise control over molecular architecture and properties [6, 7]. This high degree of structural tunability underpins their growing utility in molecular engineering and biomolecular mimicry [3, 6].

Peptides have emerged as a competitive therapeutic class, with 34 peptide‐based drugs approved by the U.S. Food and Drug Administration (FDA) between 2016 and 2025 [8]. In addition, peptides are increasingly incorporated into conjugate‐based platforms, including antibody drug conjugates (ADCs) and peptide drug conjugates (PDCs), where they function as targeting ligands, linkers, payloads or bioactive warheads [9, 10, 11]. Notably, hundreds of peptide candidates are currently in the development pipeline, including peptide inhibitors targeting RAS and mTOR pathways [12], with applications spanning oncology [13], antimicrobial therapy [14], antiviral [15] and other therapeutic areas.

In biomedicine, particularly in drug delivery and tissue engineering, the clinical translation of peptide‐based therapeutics remains constrained by issues such as rapid enzymatic degradation, limited stability, nonspecific distribution and inadequate biomimicry [1, 16, 17]. Traditional delivery systems and inorganic nanomaterials may further present challenges, including cytotoxicity and poor biodegradability [17]. To address these limitations, short peptide sequences can undergo spontaneous organisation into ordered nanostructures under thermodynamically favourable conditions, a process known as peptide self‐assembly [6, 18, 19]. This process is governed by cooperative noncovalent interactions that drive the formation of supramolecular architectures [20]. These assemblies typically occur at the nanoscale and exhibit distinct, tuneable physical, chemical and biological properties [16, 21]. This self‐assembly behaviour is central to the formation of critical biological structures, including phospholipid bilayers, as well as mediating key molecular recognition events such as ligand–receptor interactions [22, 23, 24]. Self‐assembly is a hierarchical process, progressing through defined structural stages prior to attaining its final conformation [25]. In particular, the formation of secondary structural elements, most notably α‐helices and β‐sheets, represents a crucial intermediate stage that dictates subsequent intermolecular interactions and higher‐order organisation [26]. As a result, self‐assembled peptide nanomaterials have gained considerable attention as versatile platforms in nanotechnology, materials science and particularly biomedical applications [16, 25, 27].

Peptide‐based self‐assembling nanomaterials offer a range of advantageous properties, including ease of synthesis, high structural tunability, low immunogenicity and an intrinsic ability to mimic biological architectures [3, 16]. Upon self‐assembly into nanostructures such as nanofibres, hydrogels and nanotubes, these materials can enhance the stability of encapsulated therapeutics, enable controlled and sustained drug release and provide extracellular matrix‐like environments that support cellular adhesion, proliferation and signalling [16, 28, 29, 30]. Their nanoscale dimensions further facilitate efficient interactions with biological membranes while minimising immune activation and cytotoxicity, thereby improving their suitability for in vivo applications [31]. As fundamental building blocks of proteins, peptides also exhibit exceptional structural adaptability, allowing them to organise into a diverse range of nanostructures and hydrogel systems with applications spanning biomedical, cosmetic and industrial fields [11, 32, 33, 34, 35]. Collectively, these secondary structural motifs establish the fundamental framework governing peptide folding, self‐assembly and function, thereby dictating the physicochemical properties and overall performance of peptide‐based systems. In turn, these characteristics underpin the versatility of peptide self‐assembled nanomaterials, positioning them as a highly adaptable and promising platform for applications in drug delivery and regenerative medicine [3, 16].

This review aims to summarise the key driving forces and mechanisms underlying peptide self‐assembly into functional nanomaterials, and to analyse how α‐helical and β‐sheet secondary structures influence nanomaterial architecture, properties and biomedical function. It also highlights key analytical techniques used to characterise peptide self‐assembly. Furthermore, the review provides a critical evaluation of current biomedical applications of peptide‐based nanomaterials, including their roles in therapeutic cargo encapsulation and targeted drug delivery. In addition, it identifies existing limitations, translational challenges and future opportunities associated with the clinical implementation of peptide self‐assembling nanomaterials.

## 2 Self‐Assembly Driving Forces

**FIGURE 1:**
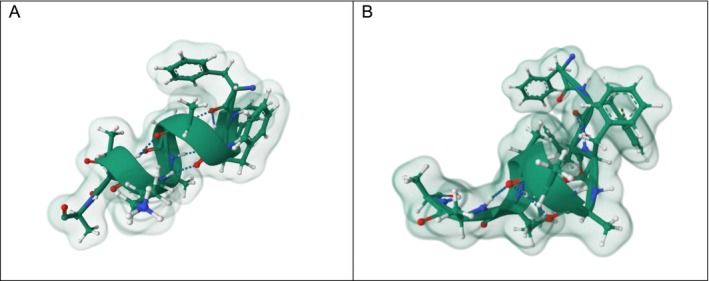
Three‐dimensional molecular model of: A. α‐helix peptide motif stabilised by intramolecular hydrogen bonds along the peptide backbone. B. β‐sheet structure formed by intermolecular hydrogen bonding between aligned peptide backbones. Hydrogen bonds are shown as dashed lines, highlighting the helical folding of the chain in A and stabilising the sheet in B. Visualised on RCSB and adapted from [37].

Peptide self‐assembly is governed by a complex interplay of intramolecular and intermolecular noncovalent interactions, including hydrogen bonding, π–π stacking, van der Waals forces, hydrophobic interactions and electrostatic forces [1]. These weak yet highly cooperative and directional interactions drive the organisation of peptide sequences into ordered secondary structures, ultimately dictating the morphology, stability and functionality of peptide‐based nanomaterials [1]. Among these, hydrogen bonding plays a dominant role due to its moderate strength and strong directionality, facilitating backbone alignment, promoting ordered molecular packing and stabilising well‐defined supramolecular architectures [1, 36]. Spectroscopic and microscopic studies consistently identify backbone hydrogen bonding as a key determinant in fibril formation and structural integrity [29]. Specifically, intramolecular hydrogen bonding stabilises α‐helical conformations, whereas intermolecular hydrogen bonding between adjacent peptide backbones promotes β‐sheet formation (Figure 1) [38].

**FIGURE 2:**
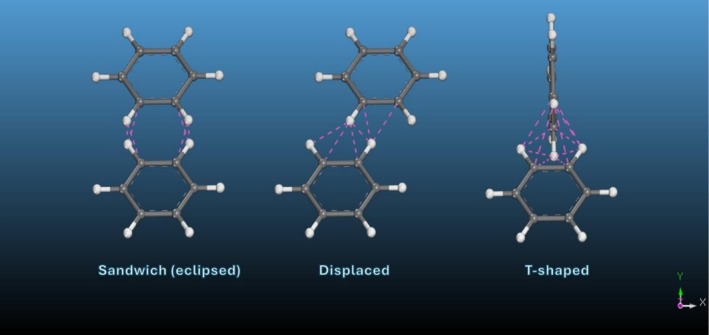
Schematic representation of π‐π stacking interactions between aromatic side chains, illustrating sandwich (eclipsed), displaced and T‐shaped stacking geometries commonly involved in peptide self‐assembly. Created with www.biorender.com [39].

In addition to hydrogen bonding, aromatic interactions play a critical role in peptide self‐assembly. Peptides containing aromatic residues such as phenylalanine and tyrosine engage in π–π stacking interactions that contribute to both nucleation and stabilisation of nanostructures in aqueous environments (Figure 2) [29]. Model systems such as diphenylalanine (FF) and aromatic dipeptides (e.g., naphthalene‐FF) demonstrate that increasing aromatic content promotes the formation of rigid nanotubes and highly ordered fibrillar assemblies [40, 41, 42, 43]. Furthermore, the incorporation of aromatic protecting groups such as 9‐fluorenylmethyloxycarbonyl (Fmoc) enhances π–π stacking interactions, thereby strengthening self‐assembly and improving structural robustness [44, 45].

Electrostatic interactions between oppositely charged side chains further contribute to structural specificity and play a key role in directing the formation of both α‐helical and β‐sheet assemblies in charged peptide systems [29]. These interactions are particularly evident in ionic‐complementary peptides, such as RADA16, which contain alternating charged residues that promote ordered hydrogel formation [46, 47]. Rational electrostatic patterning has also been shown to enhance the rheological and mechanical properties of peptide‐based materials. For example, rheological studies demonstrate that combining oppositely charged polymers, such as poly(L‐lysine hydrobromide) and poly(L‐glutamic acid sodium), yields composite hydrogels with significantly improved mechanical strength and enhanced wound‐healing performance [29].

Although individually weak, the cooperative synergy of these noncovalent interactions can generate assemblies with overall binding energies approaching those of weak covalent bonds [48]. This synergistic behaviour enables precise control over both nanoscale architecture and macroscopic material properties. For instance, in peptide hydrogel systems, hydrogen bonding is essential for initial network formation, while electrostatic and hydrophobic interactions contribute to enhanced mechanical strength, viscoelasticity and self‐healing properties [49]. Such findings underscore that functional peptide nanostructures arise from the collective action of multiple weak forces rather than any single dominant interaction [49].

The dynamic and reversible nature of noncovalent interactions allows peptide assemblies to respond adaptively to external stimuli, including changes in temperature, pH, ionic strength and light [36, 50, 51]. Additionally, structural constraints such as turn‐inducing motifs and reversible covalent interactions, including disulfide bond formation, have been exploited to further control assembly pathways and enhance structural stability. For example, cysteine‐containing peptides can form disulfide‐linked networks that reinforce supramolecular assemblies following initial noncovalent organisation [52]. This balance between attractive and repulsive forces is critical for maintaining a thermodynamically favourable low‐energy state that drives stable self‐assembly [36].

A key requirement for many peptide self‐assembling systems is reaching a critical aggregation concentration (CAC), above which nucleation and growth of higher‐order structures occur, although the definition of CAC can vary depending on the assembly mechanism [53]. This facilitates crosslinking between nanofibres, leading to the formation of interconnected supramolecular networks capable of entrapping large volumes of water and forming hydrogels [36]. The biodegradability of such hydrogels is largely governed by peptide interactions with enzymatic active sites, which are influenced by the presentation of functional groups on the nanofibre surface [54]. Preservation of peptide morphology is therefore essential to maximise noncovalent interactions, maintain structural integrity and enable sustained drug release while minimising environmental persistence and toxicity [55].

An interesting study by Castelletto and colleagues examined the self‐assembly of two KLVFF derivatives, a fragment of Aβ(16–20) [56]. Both peptides improved the viability of neuroblastoma cells exposed to Aβ(1–42) at sub‐stoichiometric concentrations. Fluorescence studies showed that NH_2_‐KLVFF‐CONH_2_ undergoes hydrophobic collapse and amyloid formation at the same CAC, whereas NH_2_‐K(Boc)LVFF‐CONH_2_ displays a two‐step process, with collapse at low concentration followed by amyloid formation at higher CAC. Terminal capping restricts fibril elongation and promotes fibre twisting, consistent with prior observations for amidated amyloidogenic peptides [56]. Boc protection of the lysine residue lowers the CAC by two orders of magnitude by reducing electrostatic repulsion and enhancing self‐assembly. The resulting fibre morphology resembles that of similarly charged systems, suggesting a balance between *C*‐terminal amidation and lysine protection effects. Grazing incidence X‐ray scattering indicates that peptide 1 forms planar lamellar structures in thin films, while peptide 2 is less ordered; both exhibit characteristic β‐sheet features [56].

Pizzella and colleagues showed that selected transthyretin‐derived peptides readily self‐assemble into soft hydrogels [57]. These systems tend to co‐assemble, reproducing key interactions that stabilise the parent protein's amyloid‐like structure [57]. Their results highlight how local structural features govern the mechanical and optical properties of the assemblies and demonstrate that amyloid fragments from PDB structures can be effectively repurposed to design new self‐assembling biomaterials [57]. They propose a pipeline combining (i) *in silico* evaluation of fragment stability via molecular dynamics simulations and (ii) in vitro validation of cross‐β assembly formation and material properties [57]. This approach underscores the potential of structurally defined protein fragments as a versatile source for developing tuneable biomaterials.

Torpey et al. investigated the mechanism of the peptide 4554 W (KDGIVNGVKA), identified via intracellular library screening as an inhibitor of α‐synuclein (aSyn) aggregation and toxicity [58]. NMR studies showed that 4554 W preferentially binds partially aggregated aSyn, with binding increasing over time. The peptide also undergoes deamidation of its central asparagine on a timescale comparable to aSyn aggregation, which enhances its association with the protein [58]. Functionally, 4554 W reduces fibril formation across several Parkinson's disease–associated aSyn mutants. While it does not decrease ThT fluorescence of pre‐formed fibrils, TEM reveals reduced fibril length, suggesting morphological remodelling rather than disassembly [58]. These findings indicate that targeting partially aggregated species may be a promising therapeutic strategy, potentially enabling a single peptide‐based approach effective against multiple aSyn variants and highlighting peptide therapeutics as viable modulators of aggregation and cytotoxicity [58].

## 3 Secondary Motifs

At the structural level, α‐helices and β‐sheets represent the primary secondary motifs underpinning peptide self‐assembly. In α‐helical structures, the polypeptide backbone adopts a compact right‐handed coil stabilised by intramolecular hydrogen bonds between backbone N–H and C=O groups, with approximately 3.6 amino acids per helical turn [59]. Typically comprising 10–15 residues [60], α‐helical assemblies can further associate into coiled‐coils, helical bundles and cylindrical nanostructures [61, 62, 63]. This α‐helix is stabilised by intramolecular hydrogen bonds between residues spaced four amino acids apart, forming a compact, often amphipathic structure with outward‐facing side chains [60]. These systems tend to exhibit greater conformational flexibility and dynamic behaviour, making them particularly suitable for responsive hydrogels and surface coatings [64, 65]. However, compared to β‐sheet assemblies, α‐helical structures often display reduced mechanical strength, necessitating strategies to enhance their stability and rheological performance [66].

In contrast, β‐sheet structures arise from intermolecular hydrogen bonding between extended peptide strands, forming rigid, highly ordered fibrillar networks [59, 60, 67]. These assemblies, typically involving strands of 3–10 residues [60], exhibit superior mechanical robustness and are widely utilised in drug delivery systems, tissue scaffolds and controlled release platforms. They are also closely associated with pathological aggregation processes in neurodegenerative diseases such as Alzheimer's disease [68, 69]. A β‐hairpin represents a related motif, comprising two antiparallel β‐strands connected by a short loop or turn region [70].

Importantly, both α‐helical and β‐sheet assemblies are capable of forming higher‐order supramolecular architectures, including nanotubes, nanospheres, nanobelts, twisted ribbons, micelles and fibrous plate‐like structures [71, 72, 73, 74, 75, 76]. These architectures can encapsulate therapeutic agents and support cell growth, with their specific morphology dictated by peptide sequence and environmental conditions [77]. Even minor modifications to amino acid composition can result in significant changes in physicochemical and biological properties, highlighting the high degree of tunability inherent to peptide‐based systems [78, 79].

Random coil regions, which lack defined secondary structure, introduce conformational flexibility and facilitate dynamic interactions within peptide systems [80]. Often located at terminal regions, these disordered segments contribute to functional adaptability and are frequently analysed using computational models to predict peptide behaviour and interaction profiles [81, 82].

## 4 Analytical Methods for Peptide Self‐Assembly

### 4.1 CD Spectroscopy

CD spectroscopy is widely used to characterise the secondary structures of peptides, which are critical for the formation and stability of self‐assembling hydrogels [83]. Distinct spectral features enable identification of α‐helices (negative peaks at 208 and 222 nm), β‐sheets (negative peak at ~217–218 nm and positive peak at ~195–198 nm) and random coils (negative peak near 198 nm) [83]. CD can also monitor real‐time structural transitions and assess thermal stability.

### 4.2 Fluorescence Spectroscopy

Fluorescence spectroscopy probes the luminescence and structural properties of peptide hydrogels [84]. Changes in emission intensity can indicate intermolecular interactions such as π–π stacking, which often quenches intrinsic fluorescence [84]. Additionally, dyes such as Thioflavin T (ThT) are used to detect β‐sheet‐rich structures, showing enhanced fluorescence around 485 nm [85].

Pyrene fluorescence spectroscopy is widely employed as a sensitive method to determine the CAC in self‐assembling systems. Pyrene, a polycyclic aromatic hydrocarbon composed of four fused benzene rings and lacking functional substituents, exhibits environment‐sensitive fluorescence properties that make it an effective probe for monitoring aggregation behaviour [86]. The technique is based on changes in the pyrene emission spectrum, particularly the intensity ratio of its vibronic peaks, which reflects the polarity of its microenvironment. As peptide or polymer concentration increases and aggregation occurs, pyrene preferentially partitions into hydrophobic domains within the assemblies, resulting in measurable spectral shifts. These changes enable precise determination of the CAC, as well as insights into aggregation number and the formation of hydrophobic cores within self‐assembled nanostructures [86].

### 4.3 Infrared (IR) Spectroscopy

IR spectroscopy identifies functional groups and provides insight into peptide secondary structures via amide bands. The Amide I region is particularly informative, with β‐sheets typically showing peaks near 1610 cm^−1^ and α‐helices near 1638 cm^−1^ [87, 88].

### 4.4 Nuclear Magnetic Resonance (NMR) Spectroscopy

NMR spectroscopy enables detailed analysis of peptide self‐assembly through chemical shift changes and intermolecular interactions. H NMR and 2D NOESY experiments reveal spatial proximity between residues and provide evidence of molecular packing [89]. Enhanced negative NOE signals indicate tighter supramolecular organisation and reduced molecular mobility during self‐assembly [89, 90].

## 5 Peptide‐Based Nanomaterials

**FIGURE 3:**
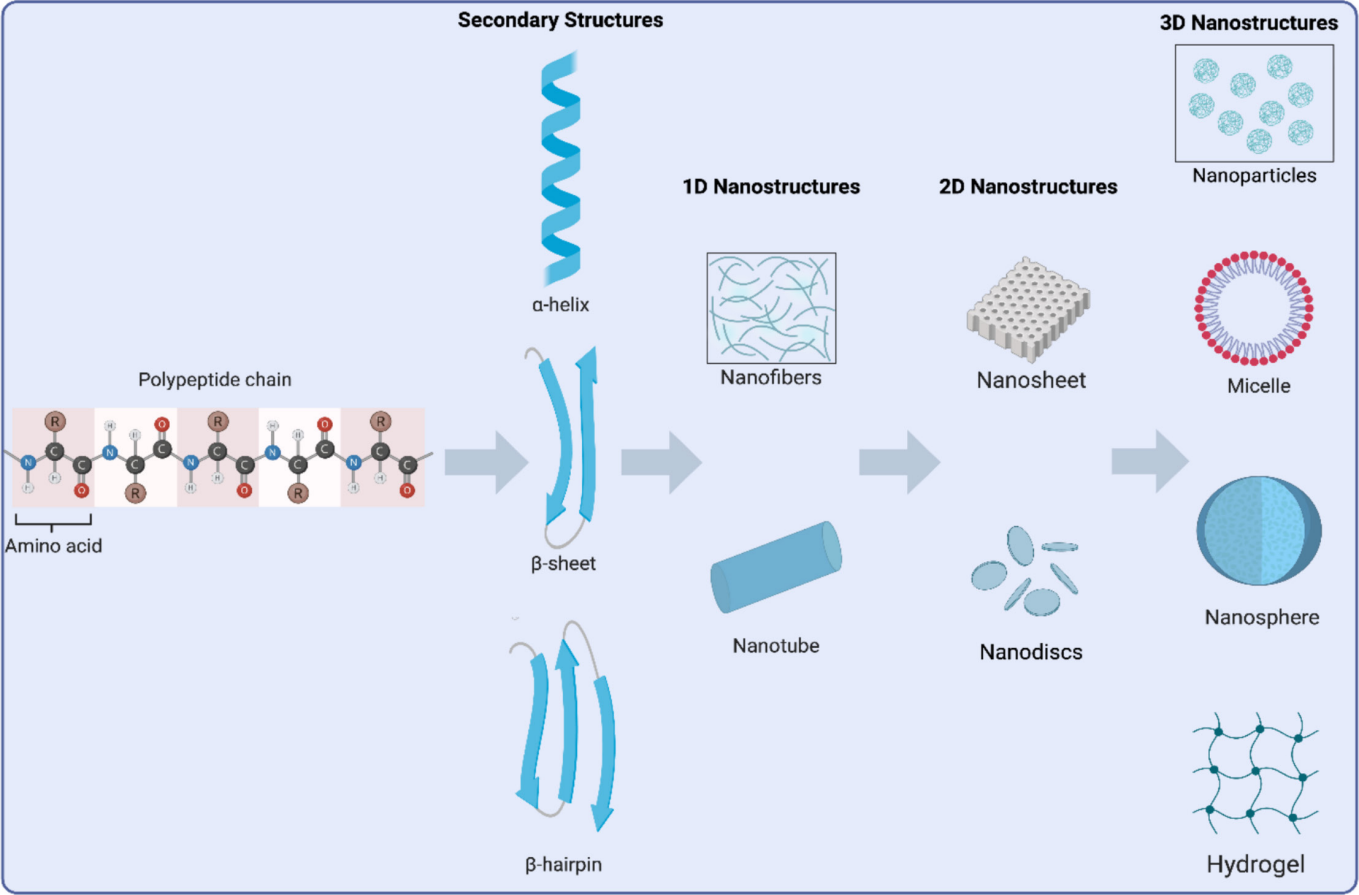
Schematic illustration of hierarchical peptide self‐assembly. Individual amino acids polymerise into polypeptide chains, which fold into defined secondary structures. These structures guide the formation of 1D nanostructures, which can laterally assemble into 2D nanomaterials and further cross‐link into 3D networks such as hydrogels. Illustration generated using www.biorender.com [39].

Peptide self‐assembly gives rise to a diverse array of nanostructures, ranging from one‐dimensional (1D) fibres and nanotubes to two‐dimensional (2D) nanosheets and three‐dimensional (3D) supramolecular networks such as hydrogels. The resulting morphology is highly dependent on peptide sequence, adopted secondary structure and environmental conditions, which collectively govern the balance of intermolecular interactions and assembly pathways (Figure 3) [16].

1D nanostructures, including nanofibres, fibrils, nanotubes and nanoribbons, arise when intermolecular interactions are anisotropic and favour extension along a single axis [16, 91]. Both α‐helical and β‐sheet motifs can give rise to such elongated architectures, albeit via different mechanisms. α‐helical peptides typically assemble through coiled‐coil interactions or amphiphilic helix packing. For example, Daniel et al. observed that amphiphilic α‐helical peptides progressively assembled into filamentous networks over several days, highlighting the role of hydrophobic interactions and helix–helix association in driving axial growth [92].

In contrast, β‐sheet and β‐hairpin peptides elongate primarily through intermolecular backbone hydrogen bonding, often reinforced by hydrophobic clustering or electrostatic complementarity [59]. These interactions promote the formation of extended fibrillar or ribbon‐like structures, which may further aggregate into amyloid‐like fibres or higher‐order assemblies depending on environmental conditions [59]. Ionic‐complementary peptides such as RADA16‐I exemplify this behaviour; alternating charged and hydrophobic residues facilitate ordered β‐sheet stacking, yielding stable nanofibres as confirmed by atomic force microscopy (AFM) and transmission electron microscopy (TEM) in studies by Dzierżyńska and colleagues [46]. These examples underscore how secondary structure directs 1D assembly pathways, forming the structural basis for higher‐dimensional architectures [16].

MAX1 (H‐VKVKVKVKVDPPTKVKVKVKV‐NH_2_) is a 20‐residue synthetic β‐hairpin peptide designed to form stimuli‐responsive hydrogels through valine/lysine‐driven β‐sheet assembly [93]. In low ionic strength or acidic conditions, it remains unfolded and soluble, but under physiological or basic conditions it folds into a β‐hairpin that rapidly self‐assembles into a hydrogel network [93]. The resulting material forms a rigid, shear‐thinning and self‐healing matrix that quickly recovers after mechanical stress, making it attractive for injectable applications [93]. Overall, MAX1 and related systems demonstrate how coupling intramolecular folding with supramolecular assembly enables the design of responsive peptide hydrogels with tuneable macroscopic properties [93].

2D nanostructures, such as nanosheets and nanoribbons, emerge when lateral interactions between peptide assemblies are favoured over unidirectional growth [94, 95]. For instance, Kong and coworkers demonstrated that GNNQQNY peptide forms ribbon‐like 2D structures on mica and highly oriented pyrolytic graphite (HOPG) surfaces, with solvent conditions significantly influencing morphology; pure water produced irregular ribbons, whereas modified solvent compositions enhanced lateral alignment and structural uniformity, as observed by AFM imaging [96]. In a related study, β‐sheet‐forming peptides assembled into well‐defined nanosheets in ethanol/water mixtures, where increasing ethanol content promoted lateral β‐sheet stacking and the formation of large, planar peptide nanosheets [94]. These structures were subsequently functionalised with gold nanorods for applications in photothermal tumour therapy [94]. Such findings highlight the importance of controlled lateral packing in directing 2D assembly and enabling functional surface architectures [16].

3D peptide nanostructures, particularly hydrogels, arise from the hierarchical organisation and cross‐linking of 1D fibrils or 2D sheets into interconnected networks [16, 97]. These networks may form spontaneously through noncovalent interactions or be reinforced through covalent or enzymatic cross‐linking strategies. For example, Dzierżyńska and colleagues demonstrated that functionalised RADA16‐I peptides form hydrogels via β‐sheet‐driven nanofibre assembly, followed by spontaneous aggregation into a three‐dimensional network [46]. These hydrogels provide highly hydrated, porous matrices capable of encapsulating bioactive molecules, supporting cell adhesion and promoting tissue regeneration [46]. However, inherent limitations such as low mechanical strength and limited control over drug release often restrict their practical application.

To address these challenges, Wang et al. developed enzymatically cross‐linked hydrogels based on Fmoc‐GFYY peptides, where tyrosine residues were cross‐linked using horseradish peroxidase and hydrogen peroxide, resulting in denser networks with enhanced mechanical properties and improved drug release profiles [98]. Similarly, Al Musaimi and coworkers demonstrated that increasing peptide concentration in elastin‐derived hydrogels produced more robust nanofibrous networks, leading to reduced permeation rates of tetrapeptide‐21 across porcine skin over 72 h and enabling sustained drug delivery [26, 37].

Marin and colleagues demonstrated that a simple tripeptide can self‐assemble into nanocomposite hydrogels in the presence of nanodiamonds (NDs) without disrupting gelation kinetics or peptide assembly [99]. While NDs do not interfere with hydrogel formation, they significantly enhance viscoelastic properties and increase the elastic modulus of the resulting material [99]. This approach establishes a platform for ND‐based nanocomposite hydrogels with improved mechanical performance and functional versatility [99]. In particular, the intrinsic properties of nanodiamonds, including luminescence and near‐infrared responsiveness, open avenues for applications in biosensing, imaging, targeted drug delivery and tissue engineering [99].

Sugiura and coworkers reported that Fmoc‐modified taurine (Fmoc‐Tau) acts as a simple supramolecular hydrogelator, self‐assembling into fibrous networks under physiologically relevant aqueous conditions. The resulting hydrogels form biocompatible structures without requiring harsh processing [100]. Confocal microscopy revealed that the fibrous assemblies can selectively accumulate basic fibroblast growth factor (bFGF) along their networks, suggesting potential for controlled biomolecule sequestration [100]. These findings highlight Fmoc‐Tau hydrogels as promising candidates for bioapplications, including cell culture scaffolds and growth factor–responsive biomaterials [100].

Overall, these studies illustrate how precise control over peptide sequence, secondary structure, concentration and intermolecular interactions enables the rational design of nanostructures across multiple length scales. By modulating assembly pathways from 1D to 3D architectures, peptide‐based systems can be engineered with tailored mechanical properties, structural stability and functional performance for diverse biomedical applications [16, 46, 98].

## 6 Stimulus‐Responsive Behaviour: Internal and External Drivers

Environmental conditions, encompassing both extrinsic stimuli (e.g., pH, ionic strength, temperature and solvent polarity) and intrinsic triggers (e.g., enzymatic activity), exert a profound influence on peptide self‐assembly by modulating side‐chain ionisation, electrostatic screening and the balance of noncovalent interactions [25, 36]. These factors can significantly alter inter and intramolecular forces and, consequently, dictate assembly pathways and final nanostructure morphology. For example, Wang and colleagues demonstrated that pH variation induces structural transitions in FF systems, enabling reversible switching between nanotubes, bilayers and vesicular assemblies [101]. This behaviour arises from pH‐dependent changes in the ionisation states of terminal amino and carboxyl groups, which modulate electrostatic interactions and molecular packing arrangements [101].

Similarly, ionic strength plays a critical role in regulating peptide assembly by screening electrostatic repulsion and promoting ionic cross‐linking. Zhang and coworkers reported that increasing ionic strength facilitates the assembly of β‐sheet‐forming peptides into three‐dimensional hydrogel networks by enhancing intermolecular cohesion [16, 29, 102]. Temperature is another key parameter influencing assembly behaviour, where thermal energy plays a role in modulating molecular mobility and interaction dynamics, thereby enabling precise control over nanomaterial architecture. Benkhaled and colleagues demonstrated that polymerisation‐induced self‐assembly (PISA) in polymer–peptide conjugates is highly temperature‐dependent, with rod‐like nanostructures forming at 4°C and spherical morphologies emerging at 70°C [103].

In addition to extrinsic factors, intrinsic biochemical triggers provide a powerful mechanism for directing peptide assembly. Yin and colleagues showed that phosphorylated tyrosine‐containing block copolymers initially form spherical aggregates that undergo a structural transition to sheet‐like assemblies upon enzymatic dephosphorylation by alkaline phosphatase [104]. This transformation reflects the removal of charged phosphate groups, which alters intermolecular interactions and drives reorganisation of the supramolecular network [104]. Such enzyme‐responsive systems underscore the potential for designing stimuli‐responsive biomaterials with spatiotemporal control over structure and function. These studies highlight how both intrinsic and extrinsic stimuli can be used to precisely control peptide self‐assembly and design functional nanomaterials [25, 36].

Despite the extensive characterisation of noncovalent interactions in controlled in vitro environments, peptide self‐assembly remains highly sensitive to subtle environmental fluctuations, which can significantly impact both assembly pathways and resulting nanostructures [105]. This sensitivity is exemplified by the work of Arul et al., who demonstrated that FF analogues can adopt a wide range of morphologies, including nanospheres, nanosheets, nanobelts, nanorods and nanotubes, depending on solvent polarity [106]. While such responsiveness provides a versatile platform for engineering tuneable and multifunctional nanomaterials, it also presents a major challenge for reproducibility [106]. In complex biological environments, minor and often unpredictable variations in local conditions can lead to significant deviations in assembly behaviour, thereby complicating the development of consistent and reliable formulations for translational and clinical applications [105, 106].

Nonetheless, the reversible nature of noncovalent interactions confers self‐healing properties to many peptide hydrogels, enabling them to recover structural integrity after mechanical disruption without the need for toxic chemical cross‐linkers [38]. Remarkably, such systems can retain up to 90% of their original mechanical properties following deformation, thereby prolonging functional lifespan and enhancing therapeutic efficacy [71]. This combination of structural adaptability, responsiveness to external stimuli and tuneable functionality positions peptide self‐assembly as a powerful platform for the design of advanced biomaterials for targeted and sustainable biomedical applications [78].

## 7 Structure–Function in Peptide Nanomaterials

Peptide nanomaterial function is intrinsically linked to secondary structure, with α‐helical and β‐sheet motifs playing a central role in dictating both assembly behaviour and emergent material properties. These structural elements not only direct the formation of 1D, 2D and 3D architectures but also govern key functional attributes, including mechanical stability, molecular recognition and responsiveness to environmental stimuli [16, 107]. Such structure‐dependent properties underpin the broad applicability of peptide‐based nanomaterials in areas such as drug delivery, tissue engineering, immunomodulation and biosensing [107]. The following subsections examine how α‐helical and β‐sheet motifs give rise to distinct functional behaviours and enable diverse biomedical applications.

### 7.1 α‐Helical Peptide Nanomaterials

Short α‐helical (coiled‐coil) peptides can be rationally designed to self‐assemble into highly ordered nanostructures, including nanotubes, through the oligomerisation of helical segments [108]. For example, Nambiar and coworkers demonstrated that a trimeric peptide derived from the GCN4 leucine zipper motif forms well‐defined hollow nanotubes with internal cavities capable of selectively encapsulating negatively charged molecules, such as fluorescein‐labelled dextran, highlighting their potential for targeted drug delivery applications [108]. Notably, nanotube formation and stability were found to be buffer‐dependent, with optimal assembly observed in phosphate‐buffered saline (PBS), where physiological ionic strength stabilised trimeric coiled‐coil interactions and enabled reproducible control over nanotube dimensions and cargo selectivity [108]. In Al Musaimi's group, second‐generation elastin‐derived peptides (EDP‐4 and EDP‐5) were engineered by substituting lysine (K) in EDP‐1 with negatively charged aspartic acid (D) and glutamic acid (E), respectively [37]. These modifications alter the net charge, promoting enhanced gelation and increased hydrogel rigidity [37]. The improved performance is attributed to the negatively charged side chains, which stabilise the helical conformation through electrostatic interactions and reduce disruptive interactions with the peptide backbone [37]. Consequently, these design changes are hypothesised to improve the sustained‐release capabilities of the resulting self‐assembled hydrogels [37].

Beyond structural assembly, α‐helical peptide systems can also exhibit stimulus‐responsive behaviour. As discussed in Section 6, Daniel and colleagues reported that amphiphilic polycationic peptides adopt helical conformations that gradually assemble over a five‐day incubation period into entangled nanofibre networks [92]. The assembly process proceeds through intermediate protofibrils to mature filamentous structures, as confirmed by ligand‐binding assays using 8‐anilinonaphthalene‐1‐sulfonic acid (ANS) and Congo Red (CR) [92]. Importantly, the introduction of curcumin disrupted fibril formation, with TEM revealing a transition from organised fibre networks to dispersed nanoparticulate structures [92]. This ligand‐sensitive behaviour demonstrates that α‐helical assemblies can be engineered as responsive biomaterials, where external molecules modulate self‐assembly pathways, offering opportunities for controlled assembly and disassembly in drug delivery and adaptive scaffold systems.

Additionally, the structural flexibility and modularity of α‐helical peptides enable the development of hybrid nanomaterials functionalised with bioactive motifs, such as cell‐adhesion ligands or targeting sequences [105]. Modular design strategies that integrate self‐assembling α‐helical domains with biologically active sequences have been shown to produce functional fibrillar hydrogels. For instance, Dranseiki and colleagues demonstrated that varying the number and spatial arrangement of self‐assembling and cell‐binding domains allows fine‐tuning of hydrogel mechanical properties and bioavailability, thereby supporting the growth and differentiation of cell types such as mesenchymal stem cells [109].

Furthermore, Vilela‐Picos and coworkers have developed antimicrobial peptides derived from nanotube‐forming cyclic peptides of alternating chirality, whose amphipathic and cationic properties promote selective interaction with anionic phospholipid‐rich membranes [110]. This interaction induces the formation of supramolecular assemblies capable of disrupting endosomal membranes, thereby enhancing nuclear delivery of therapeutic agents while bypassing efflux‐mediated resistance [110]. Cyclic peptide–doxorubicin conjugates exemplify this strategy, enabling targeted delivery to drug‐resistant cancer cells [110]. Using complementary techniques, they demonstrated that these amphipathic conjugates self‐assemble into nanotubes, bundles and two‐dimensional structures, preferentially in the presence of anionic membranes, consistent with the elevated phosphatidylserine exposure in cancer cells [110]. These conjugates modify cellular uptake and trafficking, promoting endocytosis and facilitating endosomal escape, ultimately enabling both the peptide and drug to reach the nucleus [110]. This results in a synergistic enhancement of doxorubicin cytotoxicity and sustained antitumor activity in resistant cells [110]. Overall, this supramolecular delivery approach represents a promising strategy for overcoming drug resistance, with further peptide optimisation expected to yield improved therapeutic candidates and enable combination with conventional treatments.

**FIGURE 4:**
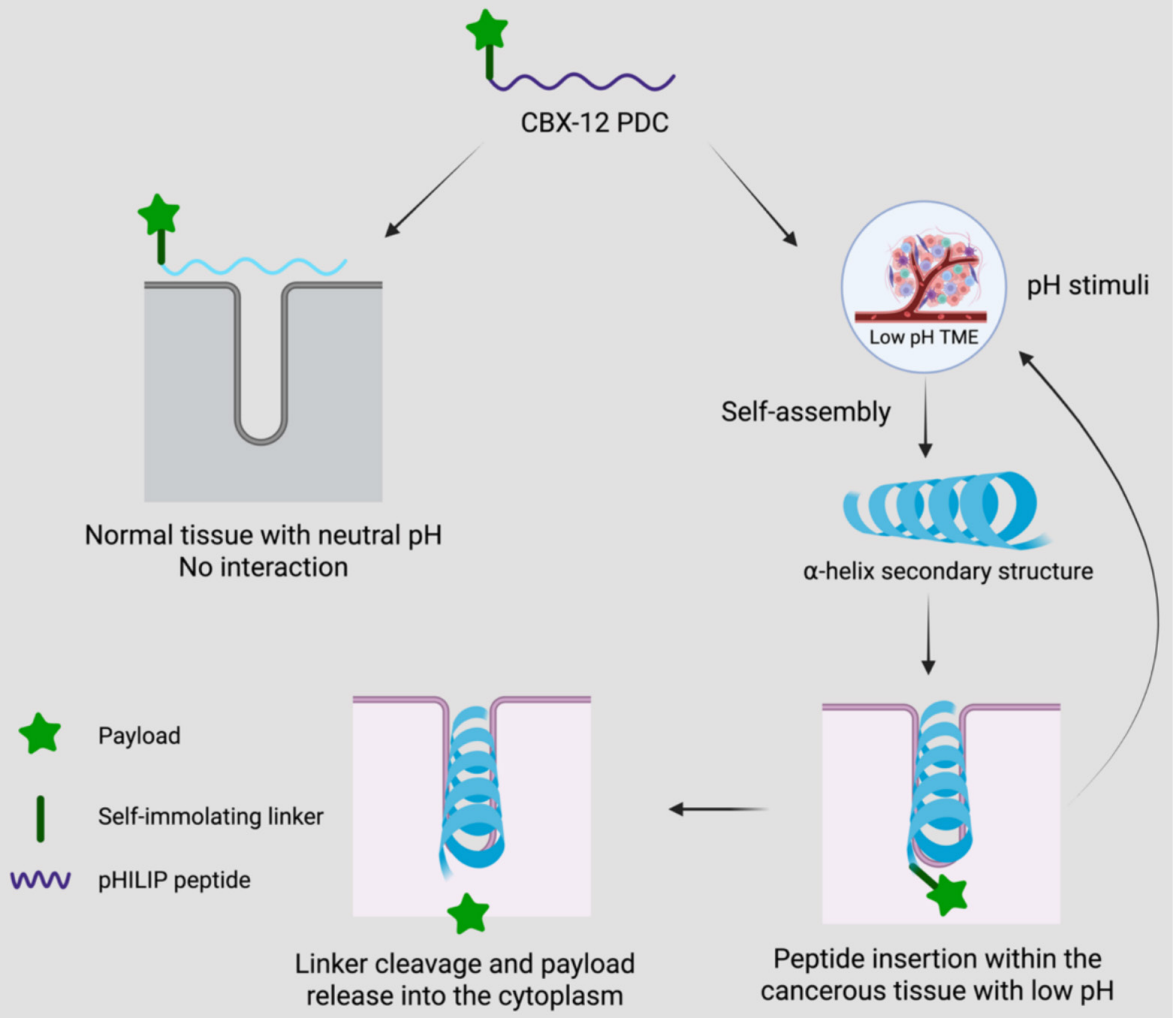
CBX‐12 mechanism of action within the tumour microenvironment (TME).

CBX‐12 underscores the clinical potential of self‐assembling peptide systems and represents a significant advance in PDCs [11]. This 26‐mer conjugate, developed by Cybrexa Therapeutics, has progressed from preclinical studies to clinical trials, demonstrating potent antitumour activity, tumour‐selective accumulation and a favourable safety profile [111]. These results supported clearance by the FDA, with Phase I trials (NCT06315491) completed in 2024 and rapid progression to Phase II following encouraging activity in platinum‐resistant ovarian cancer [111]. CBX‐12 exemplifies an innovative design that leverages pH‐triggered self‐assembly for receptor‐independent targeting. It incorporates a pH‐low insertion peptide (pHLIP; alphalex) that undergoes a conformational transition in acidic tumour microenvironments (pH 6.5–7.4), adopting an α‐helical structure that inserts into cell membranes, while remaining unstructured and inactive at physiological pH (Figure 4) [112, 113, 114]. This self‐assembly‐driven mechanism enables selective tumour delivery and provides an intrinsic safety advantage, offering a promising strategy for targeting receptor‐negative and drug‐resistant cancers [111].

Collectively, these findings highlight that α‐helical peptide assemblies are not only structurally versatile but also functionally adaptable. Their capacity for controlled self‐assembly, environmental responsiveness and biofunctionalisation positions them as promising platforms for applications in tissue engineering, regenerative medicine and targeted therapeutic delivery [105, 109].

### 7.2 β‐Sheet‐Based Peptide Nanomaterials

β‐Sheet‐based peptide nanomaterials readily self‐assemble into highly ordered fibrillar networks through extensive intermolecular backbone hydrogen bonding, resulting in mechanically robust and highly hydrated hydrogels well suited for regenerative medicine applications [46]. In contrast to α‐helical systems, β‐sheet peptides assemble via extended strand alignment, which facilitates nanofibre elongation and promotes spontaneous hydrogel formation [115]. Warren and colleagues demonstrated that P11 peptides form stable hydrogels when the net peptide charge is approximately ±2, whereas deviations from this charge hinder the formation of a percolating network. Furthermore, modulation of intermolecular hydrogen bonding, such as substituting glutamine with serine residues, reduced the elastic modulus of the resulting hydrogels by several orders of magnitude, illustrating how subtle sequence variations can significantly influence mechanical properties [115]. In conclusion, increasing ionic strength or incorporating complementary charged residues was also shown to enhance network formation and stability, enabling precise tuning of hydrogel morphology and mechanics [115, 116].

Structural analyses, including the large‐scale study by DuPai and colleagues of 49,000 β‐hairpins, have shown that stability is strongly influenced by the turn sequence, which dictates local backbone geometry and hydrogen‐bonding patterns, while the amphipathic nature of the β‐strands promotes favourable side‐chain packing and inter‐strand interactions [70]. Complementary work by Xu et al. further demonstrated that repeating β‐hairpin motifs can assemble into stable single‐layer β‐sheets, where hydrophobic clustering near the turn region compensates for the absence of a conventional hydrophobic core [117].

In addition to structural robustness, β‐sheet‐based hydrogels often exhibit desirable rheological properties for biomedical applications. Ren and colleagues reported that Fmoc‐dipeptide hydrogels (e.g., Fmoc‐YL and Fmoc‐YA) display shear‐thinning behaviour and rapid recovery of storage modulus within 30 min following mechanical disruption, a property essential for injectable systems and minimally invasive delivery [116, 118]. Similarly, Zhao et al., described thermo‐responsive PEG–polypeptide block copolymers that undergo sol–gel transitions near physiological temperatures (10°C–40°C), where temperature‐induced β‐sheet formation and chain entanglement produce injectable hydrogels with storage moduli in the range of ~0.1–1 kPa, supporting their application as regenerative scaffolds [119].

β‐Sheet peptide hydrogels have also emerged as highly effective platforms for sustained drug delivery. Their tuneable, self‐assembled networks can protect encapsulated therapeutics from degradation while enabling controlled release kinetics [16]. For example, Chen and coworkers demonstrated that the β‐sheet‐forming peptide hydrogel Rqdl10 enables sustained release of trefoil factor 3 (TFF3), achieving 81%–92% release over 36 h in vitro, depending on peptide concentration [120]. This system promoted epithelial proliferation and reduced inflammation in gastric repair models, demonstrating both controlled release and therapeutic efficacy [120]. More broadly, peptide hydrogels enhance drug stability and bioavailability by shielding labile active pharmaceutical ingredients from proteolytic degradation, thereby prolonging therapeutic activity in vivo [16, 121, 122]. Additional control over release profiles can be achieved through host–guest chemistry or responsive sequence design [123]. For instance, Cao and colleagues incorporated SBE‐β‐cyclodextrin into an Fmoc‐GFFG hydrogel, resulting in 96% retention of the chemotherapeutic HCPT after 50 h and a reduced cumulative release (52.1% vs. 91.7% without cyclodextrin), demonstrating that host–guest interactions can significantly slow drug diffusion and extend release duration [123].

Ozores and colleagues developed a β‐sheet‐forming cyclic peptide capsule capable of reversible guest recognition and release [124]. The system comprises two self‐complementary α,γ‐cyclic peptides bearing Zn–porphyrin caps, which dimerise via hydrogen bonding and are linked by dynamic hydrazone bonds [124]. This assembly combines hydrogen bonding, metal coordination and dynamic covalent interactions to enable size‐selective binding of bipyridine ligands, with release triggered by hydrazone hydrolysis [124]. The design allows tuning of the internal cavity without disrupting the peptide's self‐assembly properties [124].

Beyond drug delivery, β‐sheet‐based peptide assemblies closely mimic the structural and functional properties of the extracellular matrix (ECM), making them highly suitable for 3D cell culture and tissue engineering [28]. Yuan et al. showed that cationic β‐sheet peptides of the form (KI)𝑛K co‐assemble with hyaluronan to form ECM‐mimetic hydrogels capable of supporting mesenchymal stem cell spheroid formation [28]. Notably, peptide sequence length influenced spheroid size, with (KI)5 K–HA producing significantly larger spheroids than (KI)6 K–HA, highlighting the role of sequence design in modulating cellular microenvironments [28]. Similarly, Lingard and colleagues evaluated PeptiGel systems as synthetic ECM alternatives and demonstrated that both hydrogel charge and biochemical supplementation (e.g., laminin‐111) are critical for supporting cell viability, polarity and organoid formation [125]. These findings are supported by broader analyses indicating that the self‐healing behaviour, mechanical tunability and high‐water content of β‐sheet hydrogels collectively promote cell proliferation, migration and tissue‐like signalling [29].

Importantly, β‐sheet‐based peptide nanomaterials can also be engineered to exhibit stimuli‐responsive behaviour, enabling adaptive responses to environmental or pathological cues [126, 127]. For example, Ye and colleagues identified β‐sheet‐forming peptides such as LVEFRHY that assemble into nanofibres at physiological pH 7.5 but disassemble into nanoparticles under mildly acidic conditions, pH 6.5, reflecting pH‐sensitive structural transitions relevant to disease microenvironments [128]. Similarly, Wang and coworkers developed a pH‐responsive peptide hydrogel (Nap‐FFKKK) that forms stable networks at neutral pH but releases encapsulated curcumin under acidic conditions, enhancing antibacterial efficacy against methicillin‐resistant *Staphylococcus aureus* and improving wound healing outcomes [129]. In another example, Saenz and colleagues designed histidine‐containing multidomain peptides whose assembly behaviour depends on histidine protonation state; these systems exhibited pH‐dependent nanofibre formation, drug retention and release kinetics, further demonstrating the versatility of β‐sheet‐based materials [130].

Bayón‐Fernández and colleagues reported a cyclic peptide‐based scaffold in which four nanotube‐forming cyclic peptides are linked to a tetraphenylethene (TPE) core, enabling pH‐responsive, hierarchical self‐assembly into light‐emitting 2D nanosheets [131]. The system assembles through β‐sheet interactions, π–π stacking and hydrophobic effects, while the TPE core imparts aggregation‐induced emission (AIE) for self‐reporting behaviour [131]. Structural tuning of the TPE core allows precise control over nanosheet thickness, and the arrangement of histidine residues enables enzyme‐mimetic activity upon metal coordination [131]. This versatile platform tolerates broad structural modifications and provides a general strategy for designing functional, biocompatible 2D supramolecular materials with tuneable properties [131].

**FIGURE 5:**
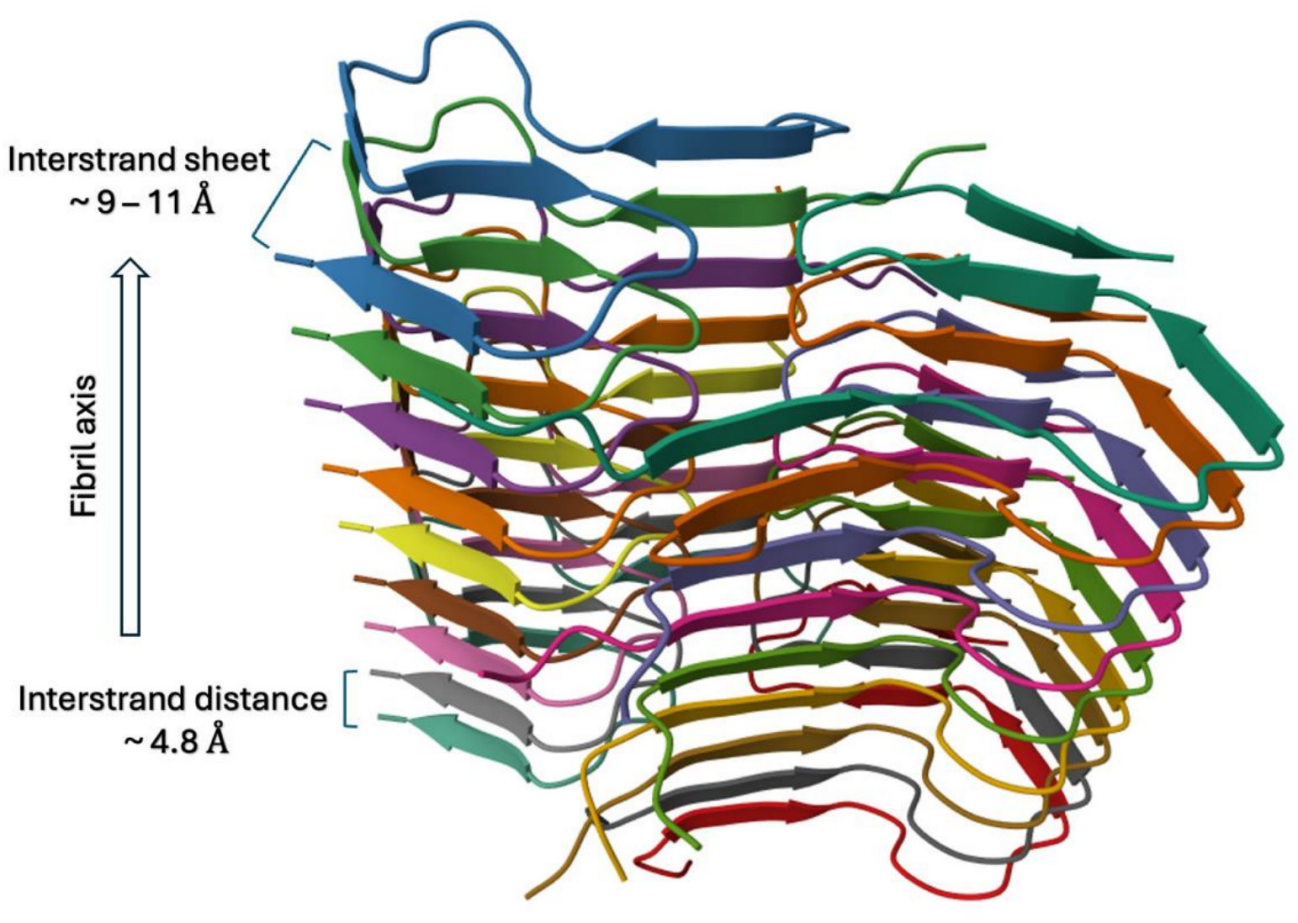
Crystal structure of monomorphic Aβ42 amyloid fibrils showing cross‐β‐sheet supersecondary structure; PDB ID 5KK3 [133].

Within β‐sheet‐rich amyloid aggregates, a small population of β‐strands forms inter‐β‐sheet connections by inserting into neighbouring sheets [132]. These cross‐β‐strand linkers constitute less than 15% of total strands, yet they contribute disproportionately to intermolecular interactions by acting as dynamic adapters that modulate inter‐sheet packing [132]. Amyloids are fibrous protein aggregates characterised by cross‐β architecture, where β‐sheets formed by identical amino acid alignments are stabilised through side‐chain interactions in a ‘steric zipper’ arrangement (Figure 5) [134]. Rather than adopting a single conformation, these linkers exist as heterogeneous ensembles, introducing structural plasticity into otherwise ordered assemblies. By engaging multiple substates, they balance rigidity and flexibility within the aggregate, enabling structural diversity and adaptability. Overall, this highlights how low‐abundance structural elements can exert outsized control over the organisation of complex self‐assembling systems [132].

Exploiting this intrinsic self‐assembly tendency, Aggeli and colleagues showed that peptides can hierarchically organise in water into tapes, twisted ribbons, fibrils and fibres as concentration increases, a process influenced by peptide chirality derived from L‐amino acids [135]. They further demonstrated that electrostatic interactions provide an additional control mechanism for assembly [135]. At specific pH windows, oppositely charged peptides can be mixed to form polyelectrolyte β‐sheet complexes (PECs), where Coulombic attraction drives spontaneous formation of fibrillar networks and nematic hydrogels [135]. These systems behave analogously to polymeric PECs but with more defined molecular and mesoscale structure, typically exhibiting 1:1 stoichiometry and robustness to pH and concentration changes [135]. Design rules include β‐sheet propensity, complementary charge patterning and sufficient residual charge to prevent flocculation, while ionic strength modulates assembly by screening electrostatic interactions [135]. These biocompatible hydrogels offer promising platforms for encapsulation, immobilisation and separation of biomolecules and cells, with tuneable supramolecular architecture dictated by peptide sequence design.

Nelson and colleagues investigated a seven‐residue segment (GNNQQNY) from the yeast prion protein Sup35, a key determinant of protein‐based inheritance and prion‐like infectivity [136]. This peptide forms amyloid‐like fibrils at micromolar concentrations, exhibiting hallmark features such as cross‐β diffraction, unbranched morphology, dye binding (Congo red and thioflavin T), cooperative nucleation‐dependent kinetics and high stability [136]. X‐ray analysis of both fibrils and related microcrystals revealed a common cross‐β spine architecture, with peptide strands oriented perpendicular to the fibril axis. Each molecule engages in an extensive hydrogen‐bonding network, including backbone hydrogen bonds and sidechain ‘amide stacks’ between Asn and Gln residues. These interactions enforce highly ordered, in‐register β‐sheet stacking and define the structural core of the aggregate [136]. The structure suggests that multiple self‐complementary segments must align to form a nucleation‐competent state, creating a significant kinetic barrier to fibril formation. However, once assembled, the resulting fibrils are exceptionally stable, with an even higher barrier to dissociation, explaining the irreversibility and persistence characteristic of prion‐like aggregates [136].

Collectively, β‐sheet peptide nanomaterials provide a highly tuneable platform for biomedical applications, combining mechanical robustness, controlled delivery, biomimetic function and environmental responsiveness. Together with α‐helical systems, they highlight the broader potential of peptide self‐assembly to generate multifunctional nanostructures for regenerative medicine and targeted therapeutics, while also underscoring challenges in stability, scalability and clinical translation.

Within these systems, cross‐β‐strand linkers act as rare but functionally critical molecular connectors that integrate multiple assembly intermediates into mature fibrils. Through dynamic interactions with heterogeneous conformational substates, they reduce structural entropy during β‐sheet assembly and help define inter‐sheet organisation. Importantly, this β‐strand donation mechanism appears specific to pathological aggregates rather than native protein folds, supporting the idea that such assemblies represent biologically relevant states in vivo.

Aggregates enriched in cross‐β‐strand linkers show increased resistance to thermal and chemical stress, enabling stability under physiological and disease‐relevant conditions. These findings refine classical hierarchical assembly models by demonstrating that low‐abundance inter‐sheet connectors are key architectural regulators of amyloid structure. Future work should focus on elucidating their mechanistic roles and implications in protein misfolding diseases.

## 8 Design Strategies and Mechanism of Peptide Self‐Assembly

Hybrid peptide assemblies represent an advanced class of biomaterials in which peptides are integrated with polymers, lipids, therapeutic agents or inorganic components to enhance structural stability, introduce stimuli‐responsiveness and impart multifunctional properties [137, 138]. Such hybridisation strategies enable the combination of the biocompatibility and self‐assembly capabilities of peptides with the mechanical robustness and tunability of complementary materials. For example, Rumon and coworkers describe polysaccharide‐based hydrogels derived from alginate, chitosan and hyaluronic acid that can be engineered into nanocomposite, injectable or stimuli‐responsive systems for applications in drug delivery and tissue engineering [139]. This work highlights how the incorporation of peptide assemblies within polymeric matrices can significantly improve the functional performance and versatility of biomaterials [139].

Minimalistic peptide motifs, particularly aromatic dipeptides such as FF derivatives, serve as important model systems for studying hierarchical self‐assembly [140]. Molecular dynamics simulations by Gu et al. demonstrated that these dipeptides spontaneously organise into rigid nanotubular architectures driven by a combination of T‐shaped π–π stacking (Figure 3), inter‐peptide hydrogen bonding and peptide–water interactions [43]. Complementary experimental studies by Baptista and colleagues further confirm that FF derivatives can form a range of nanostructures, including nanotubes, nanofibres and nanowires [40]. When incorporated into polymer matrices, these assemblies give rise to hybrid materials with enhanced mechanical strength, as well as improved electrical and catalytic properties, thereby expanding their functional applications [40].

Netti and colleagues developed a novel bioink by incorporating the Fmoc‐FF peptide into gelatin‐based formulations to address limitations of conventional gelatin (Gel) and GelMA systems, which often require UV crosslinking that can reduce cell viability [141]. While Fmoc‐FF offers strong mechanical properties, its poor elasticity and viscosity limit its direct use in 3D printing. To overcome this, the authors covalently conjugated ethylene glycol (EG) motifs to Fmoc‐FF, increasing hydrophilicity and tunability of elasticity—key parameters for printability. This strategy enabled modulation of hydrogel mechanics, where varying EG content adjusted the storage modulus, with higher peptide ratios yielding softer gels [141]. Although gelatin reduced overall stiffness, all formulations remained thermostable and highly biocompatible, supporting > 90% cell viability [141]. Overall, this work demonstrates a UV‐free, post‐printing–free approach to bioink design, producing mechanically tuneable and cell‐friendly hydrogels with strong potential for tissue engineering applications.

Diaferia and coworkers synthesised six analogues of peptide series K, replacing the *N*‐terminal acetyl group with aromatic moieties such as Fmoc or Fmoc‐FF [142]. Structural analysis showed that only the Fmoc‐modified derivatives retained gelation ability. Among them, Fmoc‐K3 formed the stiffest hydrogel (G' = 2526 Pa) and best supported cell adhesion, survival and proliferation, indicating strong potential for tissue engineering applications [142]. Hydrogel formation was driven by a balance of van der Waals forces, hydrogen bonding and π–π stacking, with β‐sheet structures adopting antiparallel strand orientation. While aromatic modification did not generally hinder self‐assembly, combining both Fmoc and FF motifs disrupted key interactions and prevented gelation, highlighting the importance of fine‐tuning competing molecular forces [142]. In vitro assays confirmed excellent biocompatibility across all hydrogels, with no cytotoxic effects observed. However, only Fmoc‐K3 provided an optimal mechanical environment for cell growth, likely due to its higher stiffness and more compact molecular packing. Overall, Fmoc‐K3 emerges as a promising biomaterial for biomedical and tissue engineering applications [142].

Amphiphilic peptides, characterised by the presence of distinct hydrophilic (e.g., lysine or glutamic acid residues) and hydrophobic (e.g., non‐polar or aromatic residues) domains, represent another important class of self‐assembling systems. Their intrinsic amphiphilicity drives the formation of diverse nanostructures, including micelles, vesicles, cylindrical fibres and fibrillar hydrogels, depending on sequence design and environmental conditions [16, 143, 144]. A notable example is reported by Fortunato and coworkers where an amphipathic peptide was conjugated to a semiconducting aromatic core, benzothieno[3,2‐*b*][1]‐benzothiophene (BTBT), resulting in the formation of a 3D fibrillar network [145]. TEM and spectroscopic analyses revealed that the assembly is stabilised through a synergistic combination of peptide backbone hydrogen bonding and π–π stacking interactions between BTBT units [145]. This hybrid system exhibited both electrical conductivity and high biocompatibility, demonstrating its potential for bioelectronic and biomedical applications [145].

Similarly, Sun and colleagues reported that designed amphiphilic peptides can assemble into β‐sheet‐rich nanofibres that further cross‐link into injectable hydrogels under physiological pH conditions [146]. These systems exhibit excellent biocompatibility and responsiveness to environmental stimuli, making them promising candidates for biomedical applications such as drug delivery and regenerative medicine, including potential use in spinal cord injury repair [146].

Peptide‐targeted MRI has important applications in cancer imaging, where peptide‐based building blocks are employed as scaffolds for supramolecular gadolinium‐containing contrast agents [147, 148].

PEGylated tetra‐phenylalanine (F4) fibres, *N*‐terminally modified with DOTA or DTPA chelating agents, have been proposed as gadolinium‐based magnetic resonance imaging (MRI) contrast agents due to their ability to form water‐soluble nanofibrous assemblies [149]. Fibre formation is primarily driven by π–π stacking interactions among phenylalanine side chains. Structural comparisons between PEGylated F4 fibres with and without DOTA modification show strong similarities, with both systems adopting antiparallel β‐sheet architectures [149]. However, DOTA‐functionalised fibres exhibit increased size and higher structural order, indicating that the macrocyclic chelator has limited impact on β‐sheet organisation [149]. This is attributed to the PEG spacer, which spatially decouples the chelating group from the aromatic core, preserving the self‐assembly behaviour while enabling stable metal coordination for imaging applications [149].

Bull and colleagues have developed MR‐active peptide amphiphiles (PAs) that self‐assemble into spherical micelles or fibrous nanostructures, enhancing T1 relaxation and enabling their use as contrast agents [150]. These peptide–amphiphile contrast agent (PACA) systems are functionalised with DOTA derivatives and can further be stabilised through disulfide crosslinking. Self‐assembly in aqueous environments increases rotational correlation time (τr), improving MR signal properties while preserving the inherent bioactivity and modularity of the peptide sequence [150]. The resulting materials combine structural tuneability with imaging capability, allowing integration of targeting functions alongside MR visibility [150]. These systems enable noninvasive, high‐resolution 3D MRI tracking of tissue‐engineered scaffolds, providing a means to monitor their localisation, migration and degradation over time in vivo [150].

Sun and coworkers developed a supramolecular peptide‐based nanodrug (SPN) that undergoes intracellular polyamine‐triggered transformation from nanoparticles into microfibres, enabling enhanced tumour accumulation and retention [151]. The system is built from a camptothecin prodrug conjugate (FFVLK‐CPT), which forms noncovalent host–guest complexes with cucurbit[7]uril (CB [7]) through Phe–CB [7] interactions and self‐assembles into nanoparticles [151]. In cancer cells with elevated spermine levels, CB [7] is dissociated, triggering a nanoparticle‐to‐microfibre transition [151]. This morphology change promotes sustained intracellular retention of camptothecin, enhancing anticancer efficacy while reducing systemic toxicity. Overall, this work introduces a stimuli‐responsive, shape‐transformable supramolecular platform for precision drug delivery and improved tumour‐selective therapy.

Wang and colleagues developed HEKMs, a tumour microenvironment–responsive nanoscale micellar system with shape‐changing and multi‐targeting capabilities for improved imaging and therapy of heterogeneous tumours [152]. The system is designed with three functional modules: (i) a recognition element targeting the epidermal growth factor receptor and human epidermal growth factor receptor 2 (EGFR–HER2) complex and mitochondria, (ii) an imaging module combining a matrix metalloproteinase‐2 (MMP‐2)–activated FRET probe for NIR fluorescence and a gadolinium component for MRI and (iii) a therapeutic payload including the pro‐apoptotic peptide KLA and the chemotherapeutic drug doxorubicin [152]. HEKMs self‐assemble into nanorods under physiological conditions, enabling prolonged blood circulation due to reduced uptake by the reticuloendothelial system [152]. Upon reaching tumour tissue, EGFR–HER2 recognition enhances cellular targeting, while MMP‐2–mediated cleavage triggers a rod‐to‐sphere transition. This shape change promotes deep tumour penetration and accumulation. The integrated system enables dual‐modal MRI/NIR imaging and synergistic apoptosis induction through mitochondrial disruption and chemotherapy [152]. Overall, this work demonstrates a programmable, shape‐transformable nanoplatform for tumour‐specific targeting, imaging and therapy, while also highlighting translational challenges associated with complex in vivo behaviour of engineered supramolecular systems.

Collectively, these studies illustrate that hybrid peptide‐based assemblies provide a versatile platform for engineering advanced functional materials. By integrating peptides with complementary molecular components, it is possible to achieve enhanced mechanical properties, improved stability and tailored functionalities, thereby expanding the scope of peptide self‐assembly for next‐generation biomedical applications [40, 137, 138, 139, 146, 150, 151, 152].

## 9 Self‐Assembly in Biomolecular Systems

Self‐assembly principles extend beyond peptides to protein‐based systems and can be leveraged for therapeutic protein delivery. By incorporating functional domains, such as receptor‐binding motifs for targeted cellular interactions and endosomolytic sequences for controlled endosomal escape, these assemblies enable enhanced delivery efficiency and improved intracellular bioavailability of protein therapeutics.

Protein self‐assembly is primarily governed by the hydrophobic effect, which defines the dominant attraction between monomers in the absence of competing forces [153]. In simplified terms, the equilibrium separation is set by the hydrophobic decay length (rH), corresponding to hydrophobic disjoining pressure [153]. When electrostatic interactions are considered alone, the separation is described by the ionic screening length (rD), as captured by the Debye–Hückel factor, which reflects the surrounding ionic atmosphere [153]. In real systems, both hydrophobic and electrostatic interactions act together, and the resulting distance depends on factors such as monomer orientation, meaning rH and rD are not universal constants [153]. Typically, rD exceeds rH and electrostatic dipole interactions shift the equilibrium distance above or below the hydrophobic baseline depending on whether they are attractive or repulsive [153]. Although steric and geometric effects can further complicate this behaviour, protein self‐assembly is inherently more complex than conventional peptide self‐assembly due to the larger size, structural heterogeneity and multidomain nature of proteins [153]. Nevertheless, protein assembly can still be effectively described using a simplified model based on hydrophobic and electrostatic pseudo‐moments, together with Brownian motion [153].

Ahn and colleagues developed a fully aqueous, genetically encoded platform for assembling micellar protein nanoparticles via electrostatic coacervation between anionic globular proteins and multi‐domain intrinsically disordered proteins (IDPs) [154]. These IDPs comprise a neutral elastin‐like polypeptide (ELP) domain and a cationic histone‐derived domain (H5) [154]. This modular system enables precise control over charge density, domain length and stoichiometry, facilitating the formation of homogeneous micellar nanoparticles with high protein payload retention under mild, biologically relevant conditions, without the need for organic solvents or covalent modification [154]. Notably, the globular proteins function both as cargo and drivers of assembly, allowing direct tuning of encapsulation efficiency [154].

Yu and co‐workers reported a self‐assembly strategy to develop a biocompatible, biodegradable protein‐based adhesive patch [155]. Hydrophobically modified fibrinogen self‐assembles into a hydrogel that is processed into a dry patch, driven by strong intra‐ and intermolecular interactions [155]. Upon application, the patch absorbs interfacial water and forms robust adhesion to wet tissue [155]. It demonstrates rapid haemostatic sealing in porcine bleeding models, including liver and arterial injuries, while maintaining biocompatibility and promoting wound healing without significant inflammation or toxicity [155]. These findings highlight how controlled protein self‐assembly can produce stable, functional biomaterials with strong clinical potential for tissue sealing and trauma care [155].

Li and co‐workers reported a DNA‐based system in which self‐assembly is tightly regulated by endogenous and exogenous triggers [156]. A palindromic DNA motif is initially inhibited by an abasic site (AP)‐linked blocking strand, preventing premature assembly [156]. Enzymatic cleavage by apurinic/apyrimidinic endonuclease 1 (APE1) removes this constraint, enabling reconfiguration and self‐assembly into extended double‐stranded DNA structures [156]. Incorporation of a photocleavable group further allows reversible disassembly under light exposure, providing precise spatiotemporal control over assembly [156].

Similarly, Cheng and colleagues demonstrated a programmable DNA nanosystem in which self‐assembly is selectively triggered within tumour environments [157]. Upon activation, DNA probes undergo a hybridisation chain reaction (HCR), forming long double‐stranded DNA polymers through a cascade of hybridisation events [157]. This stimulus‐responsive process enables controlled, in situ self‐assembly governed by tumour‐specific conditions [157].

Together, these studies highlight how DNA nanotechnology enables dynamic, stimulus‐responsive self‐assembly with precise spatial and temporal regulation, extending self‐assembly principles to nucleic acid‐based systems in complex biological environments [156, 157].

## 10 Conclusion

Peptide self‐assembly is driven by cooperative noncovalent interactions, including hydrogen bonding, π–π stacking, electrostatic forces and hydrophobic effects, which govern secondary structure formation and hierarchical organisation. α‐Helical and β‐sheet motifs critically determine architecture, stability and function, enabling 1D–3D nanostructures with tuneable mechanical and biological properties. Rational molecular design therefore links supramolecular structure directly to function, offering a route to overcome limitations of conventional therapeutics such as instability and poor bioavailability. A range of analytical techniques, including CD, fluorescence, IR and NMR spectroscopy, provide complementary insights into secondary structure, intermolecular interactions and supramolecular packing, collectively enabling precise characterisation and optimisation of peptide assemblies. Experimental studies highlight their functional versatility: β‐sheet hydrogels enable sustained protein release and tissue repair, host–guest systems modulate drug diffusion and α‐helical coiled‐coils form nanotubes for selective cargo encapsulation, confirming their therapeutic potential. This self‐assembly paradigm extends beyond peptides to proteins and DNA. Protein assembly is governed by competing hydrophobic and Debye–Hückel electrostatic interactions (rH and rD), producing complex hierarchical organisation, while DNA systems enable highly programmable, stimulus‐responsive assembly, including enzyme‐ and logic‐gated activation and reversible structural switching.

Despite these advances, clinical translation remains limited by instability, proteolytic degradation and scalability challenges. However, sequence engineering, protease‐resistant designs and AI‐guided molecular design offer promising solutions.

Overall, peptide‐based self‐assembling nanomaterials represent a highly adaptable platform for next‐generation biomedicine, integrating tuneable structure, functional diversity and programmable responsiveness for drug delivery, tissue engineering and regenerative therapies.

## Funding

The authors have nothing to report.

## Conflicts of Interest

The authors declare no conflicts of interest.

## Data Availability

Data sharing not applicable to this article as no datasets were generated or analysed during the current study.
